# Feasibility and initial efficacy of a high-intensity interval training program using adaptive equipment in persons with multiple sclerosis who have walking disability: study protocol for a single-group, feasibility trial

**DOI:** 10.1186/s13063-020-04887-x

**Published:** 2020-11-25

**Authors:** Elizabeth A. Hubbard, Robert W. Motl, David J. Elmer

**Affiliations:** 1 Department of Kinesiology, Berry College, Mount Berry, GA USA; 2 Department of Physical Therapy, University of Alabama at Birmingham, Birmingham, AL USA

**Keywords:** Multiple sclerosis, Adaptive equipment, Rehabilitation, Recumbent stepping, High-intensity interval training, Intervention, Feasibility, Exercise rehabilitation

## Abstract

**Background:**

There is considerable evidence for the efficacy of moderate-intensity continuous exercise benefitting clinically relevant outcomes in persons with multiple sclerosis (MS). However, persons with MS who have walking disability (pwMS-wd) are severely deconditioned and may achieve superior benefits by engaging in high-intensity interval training (HIIT), especially while utilizing adaptive equipment, such as recumbent arm/leg stepping (RSTEP). The proposed study will assess the feasibility of a 12-week, RSTEP HIIT program in pwMS-wd. The secondary aim will examine changes in aerobic fitness, physical activity, ambulation, upper arm function, cognition, fatigue, and depression as clinically relevant efficacy outcomes following the 12-week, RSTEP HIIT intervention.

**Methods:**

The study will recruit 15 pwMS-wd. Feasibility will be measured via process, resource, management, and scientific outcomes throughout the entirety of the research study. The secondary, clinically relevant outcomes will consist of a neurological exam, aerobic capacity, physical activity, ambulation, cognition, upper arm function, fatigue, and depression. Outcomes will be assessed at baseline (T1), midpoint (T2, following 6 weeks), and post-intervention (T3, following 12 weeks). The intervention will involve 12 weeks of supervised, individualized HIIT sessions two to three times per week. The individual HIIT sessions will each involve 10 cycles of 60-s intervals at the wattage associated with 90% VO_2peak_ followed by 60 s of active recovery intervals at 15 W, totaling 20 min in length plus 5-min warm-up and cool-down periods.

**Discussion:**

The feasibility design of the proposed study will provide experience and preliminary data for advancing towards a proof-of-concept study comparing HIIT to moderate-intensity continuous RSTEP for improving clinically relevant outcomes in a randomized control trial design. The results will be disseminated via manuscripts for publication and a report for distribution among the National Multiple Sclerosis Society.

**Trial registration:**

ClinicalTrials.gov NCT04416243. Retrospectively registered on June 4, 2020

## Background

Multiple sclerosis (MS) is a neurodegenerative disease of the central nervous system that results in clinical manifestations such as physiological deconditioning, cognitive dysfunction, and disruptions in mood [1]. One million adults are currently living with MS in the USA and upwards of 75% of those with MS report walking dysfunction that worsens with increasing disability [2–6]. Physiological deconditioning (i.e., reduced aerobic capacity) [7] is another hallmark of MS that worsens as a result of increasing disability [8], and undergirds many outcomes in MS. Accordingly, exercise training can target this cycle of physiological deconditioning and worsening disability [9] and may be a primary approach for slowing or reversing disability progression in persons with MS who have walking disability (pwMS-wd) [10]. Although there is considerable evidence for the efficacy of moderate-intensity continuous exercise (MICE) benefitting clinically relevant outcomes in persons with MS [11–16], pwMS-wd are severely deconditioned and may achieve superior benefits by engaging in high-intensity interval training (HIIT) [17–20], especially while utilizing adaptive equipment, such as with recumbent arm/leg stepping (RSTEP). RSTEP relies on similar motor activation patterns as walking [21, 22] and may increase walking performance in persons with neurological disability [21, 22], without the balance and safety risks of treadmill walking or application challenges with cycling (e.g., feet staying on pedals based on spasticity) that might affect pwMS-wd.

One systematic review summarized 7 published studies on HIIT in persons with MS [17] and identified that HIIT yielded significant improvements in cardiorespiratory fitness in all but one study [17]. Regarding studies that directly compared HIIT and MICE, the data indicated a potential superiority of HIIT for improving physiological conditioning in a time-efficient manner [17–20]. HIIT requires a shorter period of time for similar energy expenditure and may induce improvements in cardiorespiratory fitness through increases in stroke volume, maximal cardiac output, muscle capillarization, and mitochondrial content [23]. However, this evidence is specific among those with MS who have low disability engaging in cycle/arm ergometry, and the authors concluded that an investigation of HIIT in pwMS-wd is necessary as the feasibility and potential benefits are relatively unknown.

We recently published preliminary data identifying the acute effects of single sessions of high-intensity interval exercise (HIIE) as compared to MICE [24, 25]. The data indicate that a single bout of RSTEP HIIT taxed the cardiorespiratory system significantly more than MICE, yet without untoward effects on walking, gait, cognition, mood, or enjoyment [24, 25]. Those data were collected in pwMS-wd and suggested that RSTEP HIIT may be an acceptable, safe, and tolerable stimulus for chronic exercise training [26, 27]. Before moving on to a large-scale, randomized control trial (RCT), we must identify the feasibility and initial efficacy of chronic RSTEP HIIT for maximizing implementation strategies and long-term adherence with the exercise program. The feasibility data of the RSTEP HIIT program will identify if the intervention is practical through establishing the parameters of the design and to identify any potential threats to the validity of study outcomes [28]. Future iterations of the research study and design are then informed by the process, resource, management, and science outcomes, thus increasing the credibility of the next phases of research [28–31].

The proposed study will assess the feasibility of a 12-week, RSTEP HIIT program in pwMS-wd. Feasibility will be operationalized as process (e.g., recruitment, adherence, and retention rates), resource (e.g., time, space, equipment, and monetary costs), management (e.g., research training and preparation, strengths and weaknesses of expertise, and researcher capacity), and science (e.g., adverse events, participant burden, and participant feedback) outcomes [32]. We hypothesize that 12 weeks of HIIT will be feasible via process, resource, management, and scientific outcomes commensurate with other exercise interventions in multiple sclerosis (MS) [32, 33]. The study will further examine changes in aerobic fitness, physical activity, ambulation, upper arm function, cognition, fatigue, and depressive symptoms as clinically relevant efficacy outcomes following the 12-week, RSTEP HIIT intervention. We hypothesize that 12 weeks of HIIT will result in improvements in aerobic capacity, walking, upper arm function, cognition, fatigue, and depression. The clarity and strength of the proposed study stems from the primary focus on a feasibility design as opposed to conducting an initial pilot study, which emphasizes efficacy over practicality and design characteristics [28, 29, 34–37]. By documenting the study’s feasibility protocol in detail, we are able to provide valuable information to other scientists attempting to design and undertake feasibility trials before moving forward to a large-scale RCT [28].

## Methods

### Recruitment

The study will recruit 15 pwMS-wd (i.e., a PDDS score of 3–6) from areas surrounding Berry College. Berry College is located in a highly accessible, rural area in the northwest of Georgia. We will recruit from areas between Atlanta, GA; Chattanooga, TN; and Birmingham, AL. Participants will be asked to provide their own transportation to Berry College. With regard to the sample population, this study focuses on the effects of HIIT in those with MS who are ambulatory, but report walking impairments, as this represents upwards of 75% of the MS community. Although the proposed range of participants (i.e., PDDS scores of 3–6) is wide, all participants within this range report at minimum gait impairment and at maximum are primarily wheelchair users but are able to walk 25 ft in under 2 min. Using this wide range will allow us to answer our key question, while maximizing our recruitment potential in a rural, likely underserved population. We will examine medication use, symptoms of spasticity, and depression in the study results.

**Fig. 1:**
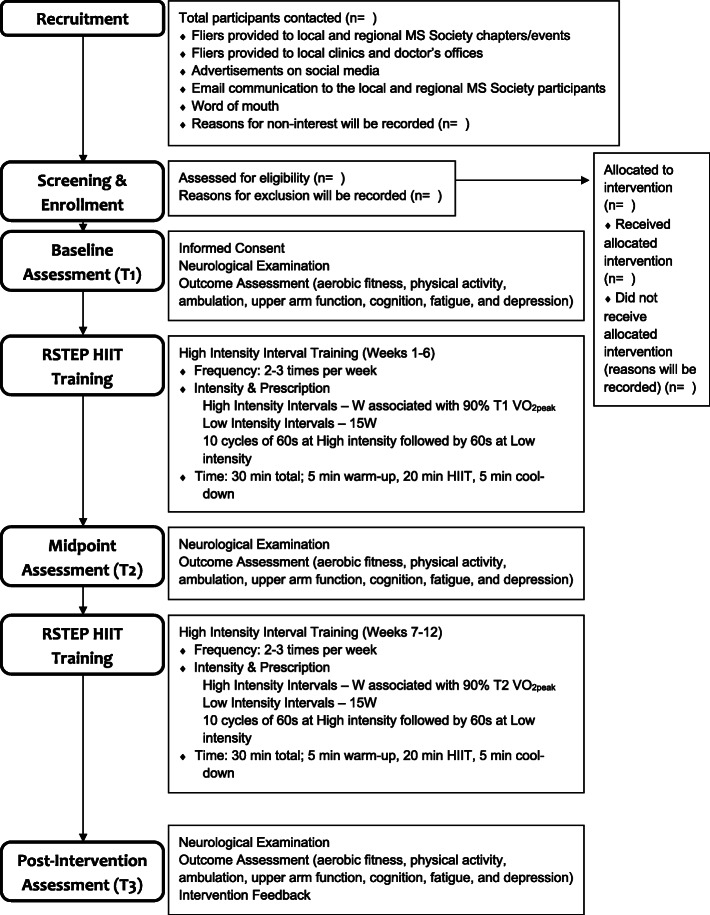
Diagram of recruitment through completion of the program. *Note*: MS=multiple sclerosis, T1=baseline assessment, T2=midpoint assessment, T3=post-intervention assessment, RSTEP=arm/leg recumbent stepping, HIIT=high-intensity interval training, VO_2peak_=peak oxygen consumption achieved at either the baseline or midpoint assessment

**Fig. 2:**
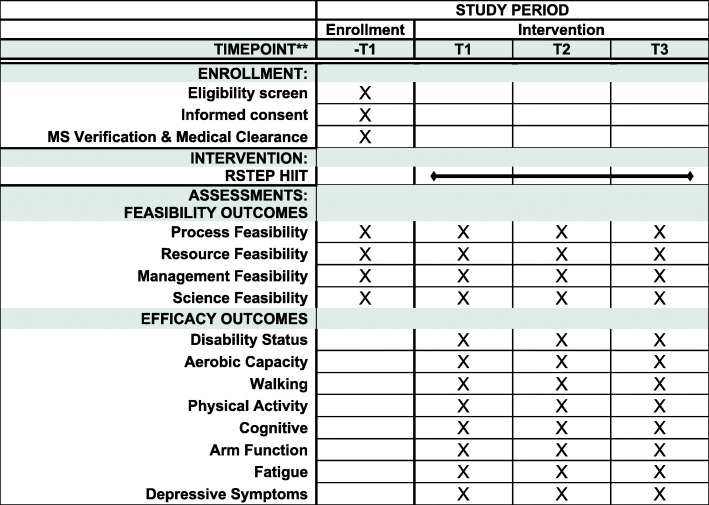
Schedule of enrollment, intervention, and assessments. *Note*: -T1=before baseline assessment, T1=baseline assessment, T2=midpoint assessment, T3=post-intervention assessment, MS=multiple sclerosis, RSTEP=arm/leg recumbent stepping, HIIT=high-intensity interval training

Recruitment will occur through fliers provided to local and regional MS Society chapters, clinics, and doctor’s offices; flyers provided at local and regional MS Society events; advertisements on social media; email communication to the local and regional MS Society participants; and by word of mouth. This study will be described as an opportunity to participate in a study testing physiological and functional responses to exercise in persons with MS. Participants will be asked to contact the laboratory by telephone or email for further information about the study and screening for inclusion. See Fig. 1 for a diagram of participant movement from recruitment through completion of the program and Fig. 2 for the schedule of enrollment, intervention, and assessments.

### Screening, eligibility criteria, and retention

#### Inclusion criteria

Participants who meet the following criteria will be included: (a) age 18–64 years, (b) a self-reported diagnosis of MS, (c) self-reported Expanded Disability Status Scale (EDSS) score < 8.0 or Patient Determined Disability Steps (PDDS) scale score ≤ 7.0, (d) relapse free in past 30 days, (e) willing and able to visit Berry College on three testing occasions and twenty-four training occasions, (f) asymptomatic status for maximal exercise testing, (g) physician approval for undertaking exercise testing, and (h) a self-reported ability to speak, read, and understand English. Participants who do not meet those criteria will be excluded from study participation. Age, self-reported MS diagnosis, relapse status, and willingness to visit Berry College’s campus will be assessed using a simple checklist. Confirmed MS diagnosis will be based on a letter from the participant’s neurologist. Disability status will be determined using the self-reported EDSS and the PDDS scale. Asymptomatic status is defined as one or fewer affirmatives on the Physical Activity Readiness-Questionnaire (PAR-Q). MS verification and medical clearance for exercise testing will be provided by the participant’s physician.

#### Screening procedure

Inclusion criteria will be assessed over the telephone or in-person by Dr. Elizabeth Hubbard, the research coordinator, or trained undergraduate research assistants. All screening personnel will undergo training prior to data collection to ensure all screening will be administered according to standardized instructions. There is no special expertise required to make inclusion/exclusion decisions. At first contact with potential participants, screening personnel will provide a description of the study using a telephone script. Potential participants will then be screened for age, diagnosis of MS, relapse status, willingness to visit Berry College on 27 occasions, disability status, asymptomatic status, and ability to speak, read, and understand English. Potential participants who meet these criteria will be sent forms for documenting the confirmed diagnosis of MS and obtaining medical clearance for exercise testing. Final decisions regarding participant inclusion will be made only once all screening materials have been received and reviewed. Elizabeth Hubbard will make final decisions on participant inclusions/exclusion. We will retain screening data for those who qualify and volunteer to participate and destroy screening data from those who do not qualify or do not volunteer.

#### MS verification and medical clearance procedure

Screened individuals who qualify for the study will be sent an information packet via email or through the US postal service. This packet will include the Informed Consent document, MS Verification form, and Physician’s Approval form. Participants may give verbal approval for study investigators to send MS Verification and Physician’s approval forms to the neurologist or physician directly via fax or confidential email. All forms need to be signed and returned by email, fax, regular mail, or in person before the participant is officially enrolled.

#### Process log

A process log will be maintained on a password-protected database to assess enrollment patterns and study feasibility. The log will contain the following demographic information: identification number, individuals’ name, and contact information. The log will also be used to track screening date, eligibility status, date enrolled, reason for ineligibility, reason for refused consent/participation, adherence, and source of subject (e.g., referral, advertising, advocacy, etc.). We will record adherence with the intervention via log books to be filled out at each completed or missed exercise session.

#### Retention plan

To promote adherence, participants will receive email-based newsletters intermittently throughout the program. These newsletters will include topics related to Social Cognitive Theory-based behavior change techniques, such as outcome expectations, self-monitoring, goal-setting, self-efficacy, and barriers and facilitators [28]. The newsletters will include instructions on behavioral techniques to improve intervention compliance and websites for more information on each of the topics included [28]. In addition to the newsletters, testing and training sessions will be scheduled based on the participant’s schedule and preferred times in order to encourage adherence and retention. Reminders will be sent via email or phone 24 h before each session. These reminders will be sent as emails or calls, based on participant preference. Participants will be also allowed to reschedule makeup sessions within each week if a regularly scheduled session is canceled. After midpoint testing, training will be adjusted based on testing outcomes to ensure all participants continue to receive appropriate treatment.

### Feasibility metrics

We adopted a feasibility study design aimed at providing experience and preliminary data for advancing to a proof-of-concept study in a RCT [32]. Process, resource, management, and science feasibility outcomes will be used to determine how the study is conducted in addition to identifying any perils or pitfalls that could lead to traps and stumbling blocks in a proof-of-concept RCT [32]. Thus, a single treatment group, repeated measures study design is proposed. No control group will be used because we specifically aim to identify the feasibility and preliminary efficacy of the HIIT exercise protocol alone in pwMS-wd [38].

Process, resource, management, and scientific metrics will be measured throughout the entirety of the research study [28, 32]. Table 1 presents the four different feasibility metrics, the specific outcomes related to each metric, how these outcomes will be monitored and assessed, and how the specific metrics are relevant to future phase II and III studies. In brief, the process feasibility outcomes assess participant recruitment and retention, which will provide optimal recruitment methods, expected recruitment rates, and refusal reasons. The resource feasibility outcomes will evaluate any research-stage-dependent time and resource issues that can occur and provide expected retention rates, barriers to participation, compliance rates, participant experience of the program and outcome assessments, suitability of the proposed setting, staff training needs, and monetary costs to conduct the research and establish areas for cost saving. The management feasibility outcomes identify potential human and data management issues and will provide detailed staff time requirements, highlight considerations for alterations, and detail recommended safety procedures. Scientific feasibility outcomes will examine the safety, burden, and treatment effect of the study. Participants’ experience, burden, and perceptions of intervention appropriateness will be assessed, and the treatment effect will be determined through calculating effect sizes for all of the clinically relevant efficacy outcomes. Effect sizes will also be used for power calculations for a proof-of-concept RCT [28, 32].

**Table 1:** Feasibility metrics, proposed methodology, and importance to future research in multiple sclerosis

Metric	Specific outcomes	Outcome assessment	Importance to future phase II and III studies
**Process** (i.e., participant recruitment, retention, and adherence assessment)	1. Recruitment and refusal rates 2. Retention and attrition rates 3. Adherence rates to study procedures, intervention attendance, and engagement	1. Flyers and presentations at local and regional support group chapters, National Multiple Sclerosis Society events, clinics and doctor’s offices, advertisements on social media, email communication, and word of mouth will be used to recruit potential participants. All contact methods with potential participants and refusal reasons will be recorded. 2. The flow of participants through the recruitment, enrollment, and intervention stages of the study will be recorded. 3. Adherence rates will be recorded via individual log books.	1. Provides information for optimizing recruitment methods, anticipated recruitment numbers, and refusal reasons. 2. Specifies focal areas for enhancing participant retention. 3. Provides target areas for optimizing adherence to the intervention.
**Resource** (i.e., time and monetary needs assessment)	1. Staff training requirements 2. Communication time and content requirements and alterations 3. Recruitment and intervention time requirements 4. Monetary research costs	1. Staff training needs will be recorded. 2. A password-protected database will be used to monitor all contact between participants and staff members. Communication type, purpose, and any alterations will be recorded 3. Detailed staff time requirements for recruitment, testing, and training will be recorded. Individual log books will be used to record individual testing and training requirements for each participant. 4. All study-related monetary costs will be recorded.	1. Details staff training requirements. 2. Establishes communication frequency and quality and emphasizes communication problems. Details communication needs and anticipated communication problems. 3. Details staff and participant time requirements for delivering and engaging in the intervention, respectively. 4. Determines monetary cost to conduct the research and establishes areas for cost saving.
**Management** (i.e., data management and safety reporting assessment)	1. IRB approval procedures 2. Time and accuracy in data collection, entry, and checking	1. All Institutional Review Board (IRB) approval procedures will be documented and will include communications between the University IRB and staff, and time from submission of IRB application to approval. 2. Data will be checked for completeness and errors. All time spent collecting, entering, and checking data will be recorded.	1. Details procedural and staff requirements for obtaining ethical approval, compliance, and monitoring. Highlights optimal safety procedures to be implemented. 2. Identifies staff time requirements and highlights considerations for alterations to ensure proper data management.
**Scientific** (i.e., safety, burden, and treatment effect assessment)	1. Adverse events, serious adverse events, and clinical emergencies 2. Participants’ experience, burden, and perceptions 3. Treatment effect	1. Adverse events, serious adverse events, and clinical emergencies will be reported and recorded. A safety plan will be enacted in the occurrence of an adverse event wherein participants will be asked to discontinue the HIIT exercise and cool-down (if currently exercising) and will be required to be free of symptoms for at least 48 h before returning to activity. 2. Participants’ experience, burden, and perceptions of intervention appropriateness will be assessed via a short survey at the end of the intervention. 3. Treatment effects will be determined via effect size and clinical meaningfulness calculations of any change in the efficacy outcome variables.	1. Establishes the safety and feasibility of the intervention and identifies considerations for alterations. 2. Determines the acceptability and highlight considerations for alterations. Identifying compliance will further allow correct conclusions to be drawn from the results. 3. Establishes data for power calculations and projected clinical impact.

#### Participant feedback

Intervention feedback will be solicited from participants upon completion of the final testing session. Participants will be asked to provide written feedback via a Likert-based survey about their satisfaction with the program, exercise leaders and equipment used, their confidence that they could continue the program, and the likelihood that they would recommend the program to others. Participants will also be asked to provide formative feedback with open-ended questions related to intervention facilitators, barriers, and suggestions for future trials. This feedback combined with the other feasibility metrics, which includes patient burden information, will be integrated into the dissemination of the results via scientific publication and through the National Multiple Sclerosis Society. Through this feedback process, patients will be involved in the proposed research process, study result dissemination, and future study design.

### Outcome assessments

#### Study testing overview

Baseline, midpoint (e.g., after 12 exercise training sessions), and post-intervention (e.g., after 24 exercise training sessions) testing will be performed at Berry College. All outcome assessors will undergo training prior to data collection to ensure that the final outcomes will be administered using standardized instructions and that the data will be collected consistently across time points. The outcome measures will be manualized and standardized. Midpoint testing will occur following 6 weeks of training. Post-intervention testing will occur following 12 weeks of training. The same specialized equipment for collecting baseline outcome measures will be used at the midpoint and post-intervention assessments. The use of a midpoint testing data point provides a temporal characterization of any outcome changes and may identify any discomforts in the initial stages of the program that might be overcome in the second half of the program. It further allows for modifications to the training stimulus based on any improvements after only 6 weeks of training. These data can ultimately inform therapists about the possible changes to expect if the intervention is successful and integrated into a clinical setting.

#### Participant characteristics

Descriptive characteristics of participants will be collected via a standard questionnaire. We will report mean (SD) for age, sex, employment status, MS disease type, and MS disease duration. Medication use could impact overall outcomes; thus, participants will complete a medication use questionnaire and data will be used as a control mechanism. Mean (SD) scores for item 7, “stiffness,” of the Multiple Sclerosis Impact Scale (MSIS) will be reported as a measure of spasticity experienced by participants [39]. The MSIS is a self-report measure comprised of 29 items with physical and psychological components. This Likert scale ranges from 1 (not at all) and 5 (extremely) and measures the impact of MS on day-to-day life in the past 2 weeks.

#### Disability status assessment

A Neurostatus-certified assessor (level C) will determine the disability status of participants through a clinically administered Expanded Disability Status Scale examination. The EDSS is a method of quantifying disability in MS and monitoring changes in the level of disability over time [40]. It is widely used in clinical trials and in the assessment of people with MS. The EDSS scale ranges from 0 to 10 in 0.5-unit increments that represent higher levels of disability.

#### Aerobic capacity assessment

Aerobic capacity will be assessed utilizing a standardized protocol for pwMS using a recumbent stepper [8]. The aerobic capacity data will serve as a manipulation check to ensure the intervention leads to significant fitness adaptations. Expired gases will be collected using a 2-way non-rebreathable valve, and oxygen consumption will be continuously measured using an open-circuit spirometry system. Participants will complete a 1-min warm-up at 15 W. The initial work rate will be set to 15 W and will be gradually increased until the participant reaches volitional fatigue. The work rate will be increased by 10 W per minute and 5 W per minute for participants who use a cane and walker, respectively; this yields an exercise test of approximately 8–12 min for these two groups who differ in disability level. HR using Polar H10 HR monitors and ratings of perceived exertion (RPE) via the Borg CR-10 RPE Scale will be recorded every minute. Participants will be asked to maintain a step rate of ~ 80 steps per minute [41]. Once the participant can no longer maintain a minimal cadence of 50 steps per minute or ends the test due to volitional fatigue, the assessment will be terminated. The highest recorded 30-s VO_2_ value will be recorded as VO_2peak_, expressed in mL/kg/min, when at least 1 of the following criteria are satisfied: (1) respiratory exchange ratio 1.10 or greater, (2) peak HR within 10 beats per minute of age-predicted maximum (i.e., 220-age), or (3) RPE 7 or greater. Peak HR, RPE, and wattage will also be recorded.

#### Walking assessments

Walking endurance will be assessed via the 6-min walk test (6MWT) [42]. Participants will be instructed to walk as far and as fast as possible for a 6-min period along a single corridor 75 ft in length with two, 180° turns. The protocol permits typical assistive device use and periods of rest within the 6-min period. The total distance traveled (ft) will be recorded. Walking speed will be measured using the timed 25-ft walk (T25FW) [8]. Participants will be instructed to walk as fast and as safely as possible along a clearly marked 25-ft long path. Using a stopwatch, one researcher will record the participant’s time (s) and another will follow alongside the participant for safety. This process will be repeated twice and scores will be averaged and converted into walking speed (ft/s).

#### Physical activity assessment

Device measurement of physical activity will occur using accelerometry via the ActiGraph GT3X+ accelerometer [43]. This model of accelerometer contains a solid state, digital accelerometer that generates an electrical signal proportional to the force acting on it along three axes. Participants will be asked to wear the accelerometer attached to an elastic belt on their non-dominant hip for 7 consecutive days. Participants will also be asked to log their wear time during those 7 days to ensure 10 h of wear time. Using the ActiLife software, sedentary, light, and moderate-to-vigorous counts of physical activity will be generated based on disability status [43].

#### Cognitive assessments

The Brief International Cognitive Assessment in MS (BICAMS) includes the oral version of the Symbol-Digit Modalities Test (SDMT), the first 5 recall trials of the California Verbal Learning Test-2 (CVLT-2), and the first three recall trials of the Brief Visuospatial Memory Test-Revised (BVMT-R) [44]. The SDMT measures information processing speed and involves matching 9 abstract geometric symbols with single digit numbers, whose pairings are located in a key. The task asks participants to voice correct numbers for unpaired symbols as quickly as possible for 90 s. The examiner will record responses, and the primary outcome of the SDMT will be reported as the number of correct responses provided in 90 s. The CVLT-2 measures verbal learning and memory. In this task, the examiner will read a list of 16 words, with 4 items belonging to 4 categories (e.g., vegetables, animals, furniture, and modes of transportation) that are randomly organized. The list will be read aloud 5 times in the same order, with each word voiced at a rate of approximately one word per second. Participants will be instructed to recall as many items as possible, in any order, following each reading of the list. The primary outcome of the CVLT-2 will be the total number of correct words identified over the 5 trials, with a maximum score of 90. The BVMT-R includes 3 trials of the examiner presenting a 2 × 3 array of abstract geometric figures in front of the participant. After 10 s, the array will be removed and the participants will be asked to draw the array as precisely as possible, with the figures in the correct location. Each drawing is scored on a 0 to 2 scale, based on figure accuracy and location. The primary outcome of the BVMT-R is the total raw score across the 3 trials with a maximum score of 36. Alternate forms of each of the cognitive tests will be used at each of the three time points.

#### Arm function assessment

The 9-hole peg test (9-HPT) will be used to assess upper arm function [45]. Participants are instructed to pick up pegs and place them one at a time into one of nine holes as fast as possible and then to remove the pegs, one at a time, with the same hand. The time (s) to complete this activity will be recorded and averaged on two trials for the dominant hand and then the non-dominant hand.

#### Fatigue assessment

Fatigue will be measured using the Fatigue Severity Scale (FSS) [46]. The FSS is a self-report measure comprised of 9 items that assess the severity of fatigue symptoms. This Likert scale ranges from 1 (strongly disagree) to 7 (strongly agree) and measures the degree of fatigue severity over the past week. All items will be summed for a final score with higher scores indicating greater fatigue severity and a maximum score of 63.

#### Depressive symptom assessment

Depressive symptoms will be measured by the Depression sub-scale of the Hospital Anxiety and Depression Scale (HADS-D) [47]. The HADS-D includes 7 items that measure depression and are rated on a 4-point scale ranging between 0 (not at all) and 3 (most of the time). Positively worded items are reverse-scored and then added with negatively worded items to create a total sum with a maximum value of 21.

### Exercise intervention

The intervention will involve 12 weeks of supervised, progressive (i.e., intensity increases after midpoint testing based on reassessment of aerobic fitness) HIIT sessions two to three times per week. Participants are allowed to choose and alter their frequency of exercise sessions per week as long as they train a minimum of two times per week. The average frequency chosen by participants will inform preferential frequency for future iterations of the study. HIIT exercise sessions will be manualized and led by exercise leaders (i.e., Drs. Hubbard and Elmer). Staff researchers will provide additional safety and monitoring support during each exercise session and assist with data collection and entry. All personnel will undergo training prior to exercise session data collection to ensure standardized sessions.

**Fig. 3:**
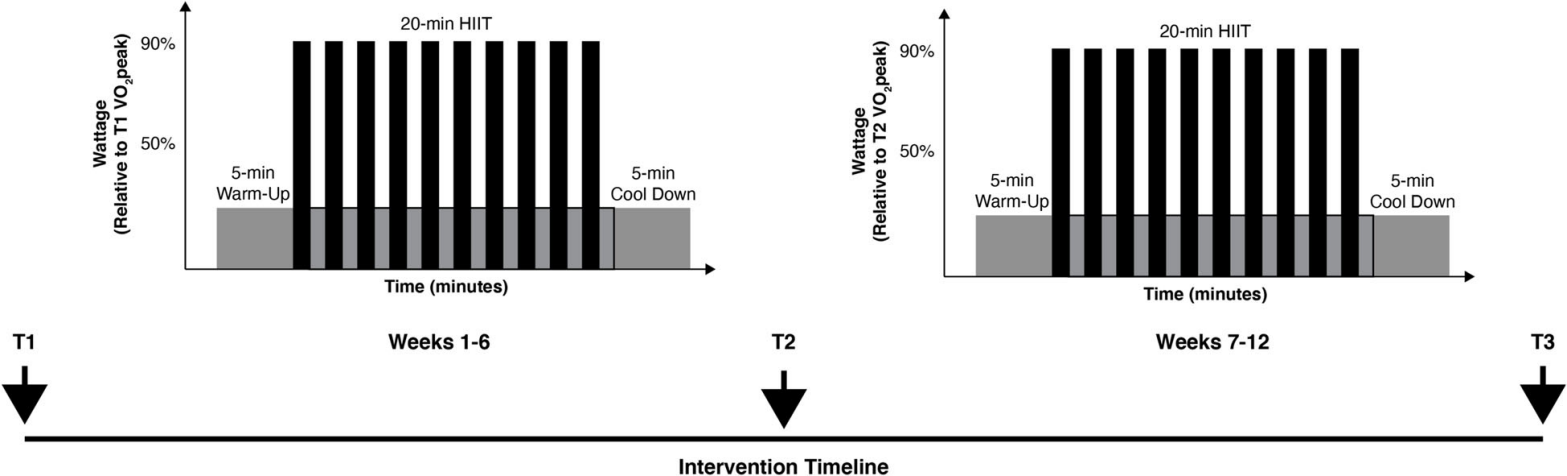
Exercise intervention progression schematics with acute exercise session protocols. *Note*: The figure is modified from Hubbard, Motl, and Fernhall [24]. T1=baseline assessment, T2=midpoint assessment, T3=post-intervention assessment, HIIT=high-intensity interval training, VO_2peak_=peak oxygen consumption achieved at either the baseline or midpoint assessment

The individual HIIT sessions will involve 10 cycles of 60-s intervals at the wattage associated with 90% VO_2peak_ followed by 60 s of active recovery intervals at 15 W, totaling 20 min in length (see Fig. 3) [24, 25]. All exercise sessions will begin and end with a 5-min warm-up and cool-down, respectively. Required power output for each interval of the exercise session will be individualized and completely automated using the SciFit FitKey software. VO_2peak_ from baseline (T1) will be used to determine exercise intensity for weeks 1–6. VO_2peak_ from midpoint (T2) will be used for determining exercise intensity for weeks 7–12. Participants will be encouraged to maintain a stepping rate of ~ 80 steps per minute throughout the exercise session [41]. Heart rate will be measured and recorded every minute to characterize the demands of the protocol. Average wattage and steps per minute will be recorded at the end of each interval minute. RPE will be measured every minute and session RPE will be measured at the end of each session using the Borg CR-10 RPE scale [48].

### Data analysis

All data will be entered, checked, and analyzed using SPSS Statistics (Chicago, IL). All data will initially be de-identified and checked for normality, outliers, errors, and missing entries. Process, resources, management, and scientific feasibility will be examined via descriptive statistics, percentage, and frequency analyses. The effect of the intervention will be determined using a repeated measures, 1-factor analysis of variance (Time) to identify significant changes over time. Multiple comparisons will be corrected using the Bonferroni correction. Disease type, medication use, symptoms of spasticity, and depression will be described and considered in the reporting of the study results. Overall effect sizes will be expressed as partial eta-squared, and small, medium, and large effects will be interpreted as values of 0.01, 0.06, and 0.14, respectively [49]. Feedback data will be reported via descriptive statistics, percentage, and frequency analysis.

We are defining clear Go/No Go Criteria for advancement to a RCT comparing RSTEP HIIT with MICE such that (1) 80% of participants (i.e., 12 participants) must complete at least 20 of the 24 prescribed sessions (i.e., 83%); this criterion is based on an expected dropout rate of ~ 15% [50]; (2) a clinically meaningful change in aerobic capacity and at least one additional outcome measure is required. Among those who successfully complete the trial (i.e., 83% of prescribed sessions), a half standard deviation improvement is necessary in aerobic fitness and at least one of the following outcomes: physical activity, walking, upper arm function, cognition, fatigue, or depression scores; this criterion is based on the universality of a half standard deviation threshold for detecting changes in health-related outcomes in chronic diseases [51, 52].

## Ethics and dissemination

### Ethical approval

Ethical approval has been obtained from Berry College’s Institutional Review Board located in northwest Georgia [protocol number 2018-19-24]. At the first baseline testing session, informed consent will be obtained via an informed consent document. Participants will be encouraged to direct any questions about the study to Dr. Elizabeth Hubbard. After reading and signing the document, participants will be officially enrolled in the study. Participants will be able to request a copy of the signed consent document following the baseline assessment. Any modifications to the protocol will be monitored via a manual of operating procedures and reported immediately to the Berry College Institutional Review Board, investigators, and trial registries.

### Safety reporting

#### Risks

The risks to participants are considered to be low and no more than typical daily life. There is little serious risk associated with the completion of the incremental exercise test and exercise sessions. The completion of maximal exercise always involves risks of death, acute myocardial infarction (i.e., heart attack), and complications that require hospitalization. There are risks of slips, trips, and falls during the assessment of functional ability. There are potential risks of becoming frustrated when completing the cognitive assessments. This will be reduced by providing clear instructions and practice tests. We will fully inform participants of the risks associated with the cognitive measures in the informed consent document. Participants will be allowed to discontinue their participation in the study at any time.

#### Protections against risk

All efforts will be made to protect against or minimize all potential identified risks. All participants will be informed of the risks associated with study participation in the informed consent document.

Risks associated with aerobic capacity outcomes and exercise training will be minimized by screening for individuals with factors placing them at increased risk for complications. All personnel will be trained in CPR and emergency lab procedures and will call 911 and Dr. Hubbard in the event of a serious adverse event as outlined in Berry College’s policies and procedures. Importantly, exhaustion, fatigue, and muscle soreness are associated with maximal aerobic exercise and strength testing, but are temporary symptoms. Participants will be encouraged to warm-up and/or stretch before any assessment of fitness or exercise session in order to minimize these symptoms. Participants will further wear clothing appropriate for exercise. Environmental regulation (e.g., use of a fan) will keep the participant cool during the sessions and avoid symptom exacerbation. Risks associated with motor function assessments will be minimized by allowing for the use of assistive devices (i.e., ankle-foot orthoses, canes, and walkers) during testing as well as having a gait belt around the participants’ waist and a research assistant within arm’s reach for stabilizing the participant in the event of a slip, trip, or fall. The testing will further be conducted in a hallway or close to a wall such that participants are able to stabilize themselves when necessary. Risks associated with the cognitive outcomes will be reduced by providing clear instructions and practice tests.

#### Reporting of adverse or serious adverse events

Participants will be instructed to notify research staff immediately when an adverse or serious adverse event occurs. Dr. Hubbard will report the AE or SAE to the Berry College Institutional Review Board. This report will include information on the event type, event severity, event expectedness, study relatedness, description of the event, and any and all steps and actions taken in response to the incident or to resolve the issue.

## Discussion

The proposed study evaluates the feasibility and preliminary efficacy of a 12-week, HIIT program using RSTEP in pwMS-wd (i.e., a PDDS score of 3.0-6.0). This work is driven by preliminary data indicating that an acute bout of HIIT taxes the cardiorespiratory system significantly more than continuous exercise, yet without sustained deleterious effects on walking, gait, cognition, core temperature, mood, or enjoyment in pwMS-wd [24, 25]. Those pilot data are from an acute or single bout of HIIT and suggest that the stimulus is acceptable, safe, and tolerable for inclusion in a chronic exercise-training program.

The proposed study will be the first to evaluate the feasibility of a 12-week HIIT program using adaptive equipment for pwMS-wd. A “feasibility study” informs the pragmatics of a RCT by defining whether an intervention can be done, should we continue with the proposed line of inquiry, and if so, how do we proceed to the RCT [53]. Process, resource, management, and science feasibility outcomes are critical to determining if an intervention is viable or not [28, 32]. Moreover, ideal participant recruitment, participant adherence, staff training, protocol execution, and data management strategies can be established. This is crucial for future research on HIIT exercise in MS. The use of a priori Go/No Go Criteria for advancement to a RCT further contributes to the strength and clarity of the proposed design. Such an initial examination of HIIT for inducing physical and functional improvements in pwMS-wd aims to reduce burdens of disability and use objective markers of progress and real-world outcomes.

This proposed study is primarily focusing on the various elements of feasibility with a secondary emphasis on the efficacy results in order to provide strength and clarity to its design [28]. The application of a HIIT paradigm among pwMS-wd is particularly relevant in clinical practice because this population is the most deconditioned of those with MS and experiences limited success with typical pharmacological methods [8, 9, 54]. Some evidence suggests that HIIT is better than continuous, moderate exercise for improving aerobic capacity, increasing ventilatory threshold, and enhancing gait economy in samples of healthy people and those with heart disease [55, 56]. The proposed HIIT protocol, which requires 20 min of exercise in a 1:1 work to rest ratio, has provided the same benefits (e.g., rapid skeletal muscle remodeling towards a more oxidative phenotype) as the traditional HIIT model in healthy adults [56]. Thus, the proposed HIIT paradigm is moving towards a much stronger stimulus for those with MS who have mobility disability.

The use of RSTEP is another innovative aspect of the study that is critical to achieving the intensity prescribed in the proposed paradigm as it removes many of the balance- and function-related issues inherent to treadmill and cycle ergometry exercise in pwMS-wd [8, 57] while utilizing the same motor activation patterns as walking [21, 22]. RSTEP has been recommended as an appropriate and viable assessment tool for evaluating cardiorespiratory fitness in neurological disorders [58], and a recent study has confirmed its efficacy for fitness assessment in MS [8]. Because graded exercise tests using RSTEP generate peak aerobic capacity values typically higher than other modalities [8], the use of RSTEP in the proposed exercise intervention will likely yield exercise prescriptions set at substantially higher workloads [8]. RSTEP further uses both the arms and the legs to generate power. Arm function is critical to the quality of life in pwMS-wd [59], yet rehabilitation specialists typically focus on ambulation. The proposed modality may have the added benefit of improving upper arm function, which would not necessarily occur in interventions using treadmills and cycle ergometers as they do not engage the arms to the same degree.

The proposed study is not without limitations. The first relates to the absence of a control group. No control group will be used because we specifically aim to identify the feasibility and preliminary efficacy of the HIIT exercise protocol alone in pwMS-wd. Once the intervention has been deemed safe and scientifically efficacious through meeting the Go/No Go Criteria set forth in the proposed study design, future iterations of the study will include a control group engaging in MICE. Another limitation to the current study is that it is lab-based and requires that participants provide their own transportation. All efforts will be made to coordinate transportation with participants, including the use of para-transit services within the immediate area. Transportation will be included as a possible reason for declining participation in the study during recruitment. Finally, the inclusion/exclusion criteria limit the generalizability of the study to those who have MS, report walking dysfunction but are able to walk 25 ft, and are under the age of 65. Although the proposed range of participants (i.e., PDDS scores of 3–6) is wide, all participants within this range report at minimum gait impairment and at maximum are primarily wheelchair users but are able to walk 25 ft in under 2 min. Using this wide range will allow us to answer our key question, while maximizing our recruitment potential in a rural, likely underserved population.

As a new rehabilitative approach, HIIT has led to significant benefits in persons with MS who have mild disability, other clinical populations, and healthy populations [18, 19, 56, 60, 61]. HIIT presents a paradigm challenge for pwMS-wd, however, as this population seemingly should not engage in high-intensity exercise—our published pilot data challenge that paradigm as will the proposed research. The proposed research may create a shift among exercise rehabilitation programs for pwMS-wd that is synonymous with the exercise revolution that occurred in cardiac care [62] by demonstrating that a HIIT intervention is feasible and provides a significant stimulus to successfully condition severely deconditioned pwMS-wd. Future studies may generate data on the effects of HIIT in pwMS-wd on motor learning capacity and possible alterations to brain structure and integrity.

## Trial status

Enrollment began on December 19, 2019. NCT04416243 (protocol version 1.2, January 21, 2020) was retrospectively registered with ClinicalTrials.gov on June 4, 2020. As of June 12, 2020, six participants have been enrolled in the study. Expected date when recruitment will be completed is May 30, 2021.

## Data Availability

Once the study is completed, only the researchers at Berry College will have access to the final dataset. Results will be submitted for publication in peer-reviewed journals regardless of the study outcome and communicated through the National Multiple Sclerosis Society at the conclusion of the study. We do not intend to use professional writers for future publications.
